# Diagnostic performance of RIPASA versus modified Alvarado score in acute appendicitis: a prospective diagnostic accuracy study from a tertiary centre in India

**DOI:** 10.1186/s12893-026-04020-w

**Published:** 2026-07-03

**Authors:** Harkirat Singh, Rajendra Kumar Yadav, Farukh Khan, Garima Agarwal, Shalu Gupta, Vinay Garg

**Affiliations:** Department of General Surgery, S.M.S. Medical College and Attached Hospitals, Jaipur, India

**Keywords:** Acute appendicitis, RIPASA score, Modified Alvarado score, Diagnostic accuracy, ROC curve, Histopathology

## Abstract

**Purpose:**

Acute appendicitis is a common surgical emergency. Clinical scores assist risk stratification where routine advanced imaging is impractical. The Alvarado score’s performance in Asian populations is debated. The Raja Isteri Pengiran Anak Saleha Appendicitis (RIPASA) score was developed for Asian settings. This study prospectively compared RIPASA and the Modified Alvarado Score (MAS) against histopathological examination (HPE) in an Indian tertiary centre.

**Methods:**

Prospective diagnostic accuracy study of 62 patients undergoing appendicectomy for suspected acute appendicitis at S.M.S. Medical College, Jaipur (January–December 2024). Pre-operative demographics, symptoms, signs and laboratory parameters were recorded; RIPASA (cut-off ≥ 7.5) and MAS (cut-off ≥ 7) were calculated. HPE was the reference standard. Outcomes were sensitivity, specificity, positive predictive value (PPV), negative predictive value (NPV) and area under the receiver operating characteristic curve (AUC) with standard error (SE) and 95% confidence interval (CI). AUCs were compared using DeLong’s test, and paired sensitivities and specificities using McNemar’s test.

**Results:**

Participants were predominantly males (71.0%); mean age 30.66 years. HPE confirmed appendicitis in 56/62 (90.3%); negative appendicectomy rate 9.7%. RIPASA ≥ 7.5: sensitivity 94.6%, specificity 66.7%, PPV 96.4%, NPV 57.1%, AUC 0.921 (SE 0.052; 95% CI 0.820–1.000; *p* = 0.001). MAS ≥ 7: sensitivity 71.4%, specificity 83.3%, PPV 97.6%, NPV 23.8%, AUC 0.821 (SE 0.083; 95% CI 0.659–0.984; *p* = 0.010). DeLong’s test showed superior discrimination for RIPASA (*p* = 0.030). McNemar’s test demonstrated higher sensitivity for RIPASA (*p* = 0.0009), with no significant difference in specificity (*p* = 1.000).

**Conclusion:**

RIPASA demonstrated higher sensitivity and better diagnostic discrimination than MAS in this tertiary surgical cohort, while MAS showed higher specificity. RIPASA may help reduce missed diagnoses in similar high-prevalence emergency settings, although further validation in broader patient populations is warranted.

## Introduction

 Acute appendicitis is one of the most common surgical emergencies worldwide, with a lifetime risk of approximately 7–8% (about 8.6% in males and 6.7% in females) [1, 2]. Although ultrasonography (US), computed tomography (CT), and magnetic resonance imaging (MRI) improve diagnostic accuracy, their routine use is often limited by availability, cost, radiation exposure (CT), and operator dependence (US). Contemporary guidance therefore recommends structured clinical risk stratification with selective imaging, rather than routine imaging for all patients [3]. Clinical assessment remains the cornerstone of diagnosis, supported by validated scoring systems to standardise bedside decision-making [4].

The Alvarado score and the Modified Alvarado Score (MAS) are widely used benchmarks [4, 5]. However, their performance appears population-dependent, and lower sensitivity has been reported in Middle Eastern and Asian cohorts [6, 7]. To address this gap, the Raja Isteri Pengiran Anak Saleha Appendicitis (RIPASA) score was developed in 2008 at RIPAS Hospital, Brunei Darussalam, for Asian populations [6, 7]. It has shown higher sensitivity and stronger overall discrimination than Alvarado/MAS in several studies and meta-analyses [6–8].

Appendicectomy remains the definitive treatment for acute appendicitis, and laparoscopic appendicectomy is preferred as it is generally associated with less postoperative pain, fewer wound complications and shorter hospital stay than open surgery [9, 10].

Delayed or missed diagnosis increases the risk of perforation and complications, whereas overdiagnosis contributes to negative appendicectomy. Therefore, accurate and timely diagnosis is essential to optimise patient outcomes and resource utilisation.

The present study aimed to compare the diagnostic performance of RIPASA and MAS for the early identification of acute appendicitis in a tertiary care setting, using histopathological examination as the reference standard.

## Materials and methods

### Study design and setting

Prospective diagnostic accuracy study at the Department of General Surgery, S.M.S. Medical College and Attached Hospitals, Jaipur, India (January–December 2024). Institutional Research Review Board and Ethics Committee approval was obtained (Ref. No. 68; dated 8 January 2024). Written informed consent was taken from all participants.

### Participants

Consecutive patients aged 15–60 years with a clinical diagnosis of acute appendicitis, admitted for appendicectomy (open or laparoscopic), were eligible. Exclusion criteria included appendicular perforation, abscess or lump, recurrent right iliac fossa (RIF) pain, incidental appendicectomy, pelvic inflammatory disease, and pregnancy. Because enrolment was restricted to patients already selected for appendicectomy, the study evaluated the scores in a high-prevalence surgical decision-making cohort rather than in an undifferentiated emergency population.

### Sample size and recruitment

The sample size was calculated using the single-proportion formula $\mathbf{n}=\left(\mathbf{Z}_\mathbf{1}-\boldsymbol{\alpha}/_2\right)^2\;\times\mathbf{p}\times\mathbf{q}\times\mathbf{d}^2$, assuming a 95% confidence level (α = 0.05; Z = 1.96). Here, p represents the anticipated sensitivity of the RIPASA score (~ 95%), derived from previously published studies reporting high diagnostic performance [11], and q = 1 − *p* = 0.05. The absolute precision (d) was taken as 7% (0.07). Based on these parameters, the calculated minimum sample size was 37. After allowing for a 10% non-response/dropout margin, the minimum required sample size was 40.

Eligible patients were enrolled consecutively during the study period. Written informed consent was obtained prior to enrolment. The final study population comprised 62 patients.

### Variables and scoring

Pre-operative demographics, symptoms, signs and laboratory data were recorded on a structured proforma. Both MAS and RIPASA were calculated for each patient. Cut-offs were prespecified: MAS ≥ 7 [12] and RIPASA ≥ 7.5 [7]. Imaging findings (ultrasonography and/or computed tomography), when available, were recorded but were not included in score calculation or used as the reference standard.

### Reference standard and outcomes

All specimens underwent histopathological examination (HPE) at the Department of Pathology, S.M.S. Medical College. Primary outcomes included sensitivity, specificity, positive predictive value (PPV), negative predictive value (NPV) and area under ROC curve (AUC) for each score using HPE as the reference standard. The secondary outcome was the negative appendicectomy rate.

### Statistical analysis

This was performed using IBM SPSS Statistics (Version 31.0, IBM Corp., Armonk, NY) and R software (R Foundation for Statistical Computing, Vienna, Austria). Categorical variables were expressed as frequencies and percentages, and continuous variables as mean ± standard deviation (SD) or median (range), as appropriate. Diagnostic performance parameters including sensitivity, specificity, positive predictive value (PPV), and negative predictive value (NPV) were calculated from 2 × 2 contingency tables with 95% confidence intervals (CI).

Receiver operating characteristic (ROC) curves were constructed and the area under the curve (AUC) with standard error (SE) and 95% CI was calculated. Direct comparison between the discriminatory ability of RIPASA and MAS was performed using DeLong’s test for paired ROC curves, and paired sensitivities and specificities were compared using McNemar’s test. A p-value < 0.05 was considered statistically significant. Reporting followed STARD 2015 recommendations for diagnostic accuracy studies. 

## Results

A total of 62 consecutive patients underwent appendicectomy for clinically suspected acute appendicitis (January–December 2024).

### Demographic and clinical profile

Participants were predominantly young adults (mean age 30.66 ± 8.94 years; median 30; range 17–53) with a male majority (44/62, 71.0%) (Table 1).

**Table 1:** Baseline demographics of participants:

Parameter(s)	Frequency (*n* = 62)	Percentage (%)
Age		
* ≤* 20 yrs	8	12.9
21–30 yrs	25	40.3
31–40 yrs	21	33.9
> 40 yrs	8	12.9
Gender		
Male	44	71.0
Female	18	29.0
Total	62	100.0

All patients reported right iliac fossa pain. Other common symptoms were migration of pain to the right lower quadrant (RLQ) in 39/62 (62.9%), nausea/vomiting in 36/62 (58.1%), and anorexia in 29/62 (46.8%). More than half presented after 48 h (34/62, 54.8%). On examination, RIF tenderness was universal; rebound tenderness was present in 42/62 (67.7%), fever in 38/62 (61.2%), Rovsing’s sign in 34/62 (54.8%), and guarding in 18/62 (29.0%). A raised total leucocyte count (TLC > 10.0 × 10^9^ cells/L) occurred in 52/62 (83.9%), and urinalysis was negative in 54/62 (87.1%).

### Histopathological diagnosis

All resected specimens of appendix underwent HPE, which confirmed acute appendicitis in 56 patients (90.3%). Across the cohort, the most common patterns were acute appendicitis (25/62) and acute appendicitis with peri-appendicitis (21/62); less frequent findings were acute on chronic appendicitis (6/62), acute necrotising appendicitis (2/62), acute necrotising appendicitis with peri-appendicitis (1/62) and acute appendicitis with reactive lymphoid hyperplasia (1/62). Reactive lymphoid hyperplasia without acute inflammation occurred in 6 patients and accounted for negative appendicectomies (negative appendicectomy rate of 9.7%) (Table 2).

**Table 2:** Clinical and laboratory parameters according to HPE outcome

Parameter(s)	HPE confirmed appendicitis (*N* = 56)	Negative appendicectomies (*N* = 6)
Age		
* ≤* 40 yrs	50 (89.3%)	4 (66.7%)
> 40 yrs	6 (10.7%)	2 (33.3%)
Gender		
Male	39 (69.6%)	5 (83.3%)
Female	17 (30.4%)	1 (16.7%)
Symptoms		
RIF Pain	56 (100.0%)	6 (100.0%)
Migration to RLQ	37 (66.1%)	2 (33.3%)
Anorexia	28 (50.0%)	1 (16.7%)
Nausea/Vomiting	33 (58.9%)	3 (50.0%)
Duration < 48 h	26 (46.4%)	2 (33.3%)
Duration > 48 h	30 (53.6%)	4 (66.7%)
Signs		
RIF Tenderness	56 (100.0%)	6 (100.0%)
Guarding	17 (30.4%)	1 (16.7%)
Rebound Tenderness	37 (66.1%)	5 (83.3%)
Rovsing’s Sign	34 (60.7%)	0 (0.0%)
Fever	33 (58.9%)	5 (83.3%)
Investigations		
Raised TLC	50 (89.3%)	2 (33.3%)
Negative Urinalysis	53 (94.6%)	1 (16.7%)

Among individual clinical and laboratory variables, Rovsing’s sign (*p* = 0.0212), raised TLC (*p* = 0.0058), and negative urinalysis (*p* = 0.0004) were significantly associated with HPE-confirmed appendicitis. RIF pain and tenderness were present in all patients. Migration, guarding, rebound tenderness and fever were more frequent with appendicitis but did not reach statistical significance.

### Diagnostic performance of scoring systems

Using prespecified cut-offs, RIPASA ≥ 7.5 classified 55/62 (88.7%) patients above threshold; MAS ≥ 7 classified 41/62 (66.1%) above threshold (Table 3, 4 and 5).

**Table 3:** Contingency table for RIPASA

RIPASA	Acute appendicitis on HPE	No Acute appendicitis on HPE	Total
≥ 7.5	53 (True Positive)	2 (False Positive)	55
< 7.5	3 (False Negative)	4 (True Negative)	7
Total	56	6	62

**Table 4:** Contingency table for MAS

MAS	Acute appendicitis on HPE	No Acute appendicitis on HPE	Total
≥ 7	40 (True Positive)	1 (False Positive)	41
< 7	16 (False Negative)	5 (True Negative)	21
Total	56	6	62

χ² test *p*-value for association between score threshold and HPE outcome: 0.001 for RIPASA and 0.014 for MAS

**Table 5:** Diagnostic performance of RIPASA and MAS versus HPE

Scoring system	Sensitivity (95% CI)	Specificity (95% CI)	PPV (95% CI)	NPV (95% CI)
RIPASA (≥ 7.5)	94.6% (85.1–98.9)	66.7% (22.3–95.7)	96.4% (87.5–99.6)	57.1% (18.4–90.1)
MAS (≥ 7)	71.4% (57.8–82.7)	83.3% (35.9–99.6)	97.6% (87.1–99.9)	23.8% (8.2–47.2)

Values are presented as percentage (95% confidence interval) unless otherwise stated. Sensitivity, specificity, positive predictive value (PPV), and negative predictive value (NPV) were calculated from 2 × 2 contingency tables, and 95% confidence intervals were estimated using the exact binomial method

### Receiver operating characteristic (ROC) analysis and comparative statistical testing

**Fig. 1:**
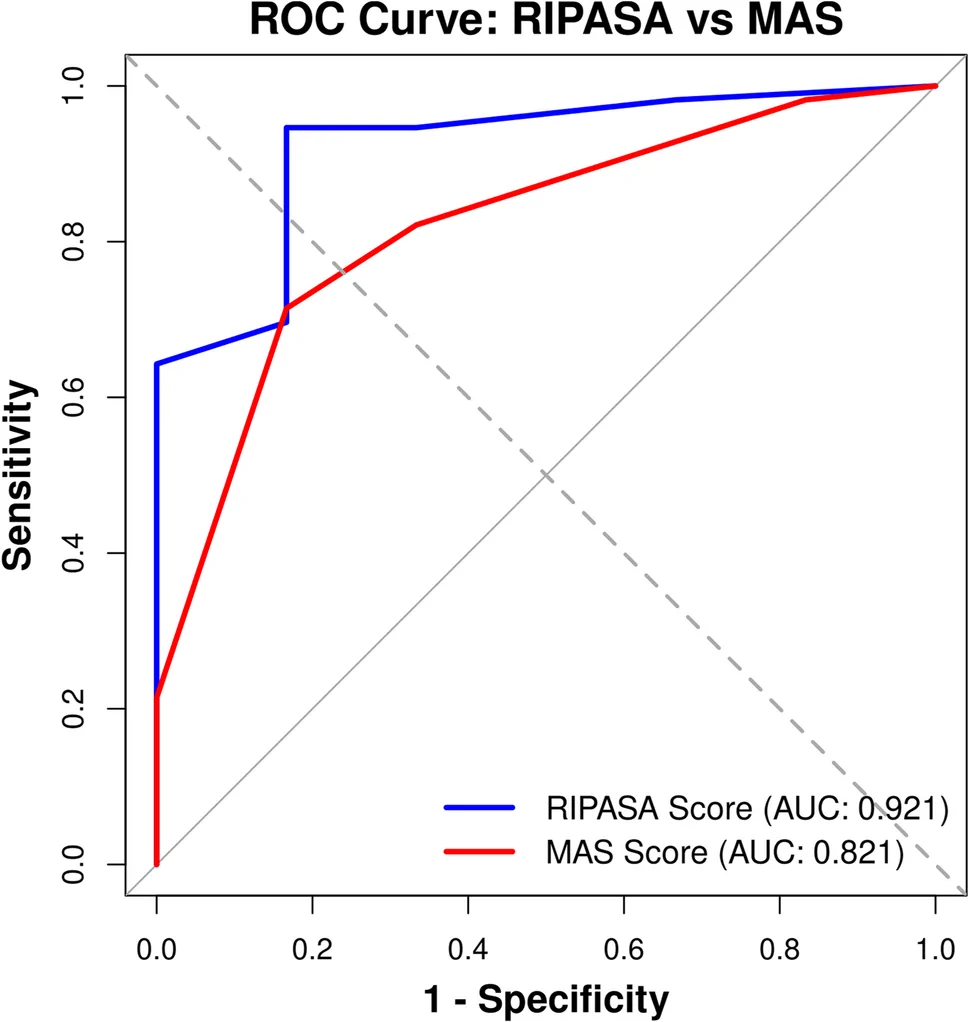
Receiver operating characteristic (ROC) curve

ROC analysis was performed with HPE serving as the reference standard. The area under the curve (AUC) for RIPASA and MAS is presented in Table 6 and illustrated in Fig. 1.

**Table 6:** AUC of RIPASA and MAS

Scoring system	Area Under Curve (AUC)	Standard Error (SE)	95% CI	*p*-value
RIPASA	0.921	0.052	0.820-1.000	0.001
MAS	0.821	0.083	0.659–0.984	0.010

ROC analysis demonstrated an AUC of 0.921 (95% CI 0.820–1.000) for RIPASA and 0.821 (95% CI 0.659–0.984) for MAS.

Direct comparison of the two ROC curves using DeLong’s test demonstrated a statistically significant difference between the AUCs (Z = 2.168, *p* = 0.030).

Comparison of paired sensitivities using McNemar’s test showed a statistically significant difference between RIPASA and MAS (*p* = 0.0009). Comparison of paired specificities showed no statistically significant difference (*p* = 1.000).

This was performed with HPE diagnosis serving as the gold standard.

## Discussion

### Summary of principal findings

In this prospective diagnostic accuracy study of operated patients with suspected acute appendicitis, RIPASA demonstrated higher sensitivity and better overall discrimination compared to MAS. MAS retained higher specificity. Both scores showed high positive predictive values, while RIPASA provided a relatively higher negative predictive value. These findings suggest that RIPASA may be more suitable for reducing missed diagnoses, whereas MAS may contribute to improving diagnostic specificity within this tertiary surgical cohort.

### Patient profile and clinical presentation

The study population reflected the classical epidemiology of acute appendicitis, with predominance of young adults (mean age 30.66 years; most between 21 and 40 years) and a male majority (71.0%), consistent with established patterns [1, 2]. Clinical presentation was typical, including right iliac fossa pain, migration of pain (62.9%), nausea/vomiting (58.1%), and anorexia (46.8%). Laboratory and examination findings such as raised total leukocyte count and positive Rovsing’s sign were consistent with established bedside predictors and components of commonly used clinical scoring systems [4, 5, 12].

### Comparison with existing literature

The diagnostic profile observed in this study—higher sensitivity and stronger discrimination for RIPASA with a trade-off in specificity, and comparatively higher specificity but lower sensitivity for MAS—is consistent with prior studies across Asian and mixed populations. Nanjundaiah et al. reported RIPASA sensitivity of 96.2% compared to 58.9% for MAS, with MAS specificity of 85.7% [13]. Shuaib et al. reported RIPASA sensitivity of 94.5% [14], and Sanjive et al. reported 97.1% [15].

MAS has consistently demonstrated higher specificity with lower sensitivity across studies. Kurane et al. reported sensitivity/specificity of 78.3%/83.8% [16], while Nanjundaiah et al. reported 58.9%/85.7% [13]. Reported MAS sensitivities vary widely—from 30.9% (Shivakumar et al.) to 90.6% (Arroyo-Rangel et al.)—reflecting differences in study populations, prevalence, and threshold selection [17, 18].

Meta-analytic evidence supports these findings. Frountzas et al. reported pooled sensitivity of approximately 94% for RIPASA compared to 79% for Alvarado-based systems, with comparable or slightly lower specificity for RIPASA [19]. Evidence from Western populations also suggests that RIPASA performs well outside Asian settings, although its diagnostic characteristics remain influenced by local prevalence and case-mix (Malik et al.) [20].

Predictive values observed in this study are concordant with prior reports. Mumtaz et al. reported PPV around 98% [21], while Goel et al. demonstrated higher NPV for RIPASA (~ 55.6%) compared to Alvarado (~ 23.3%) [22], closely aligning with the pattern observed in the present study.

### Explanations for observed patterns

The differences between the two scoring systems likely reflect their underlying design. RIPASA incorporates additional variables such as age, gender, duration of symptoms, Rovsing’s sign, and negative urinalysis, which may enhance sensitivity by capturing atypical or early presentations. In the present study, some of these variables—including Rovsing’s sign, raised total leucocyte count, and negative urinalysis—showed significant association with histopathologically confirmed appendicitis, which may partly explain the higher sensitivity observed with RIPASA. In contrast, MAS relies on a more concise set of clinical variables, which improves specificity but may increase false negatives, particularly in early or atypical presentations.

The high positive predictive values observed for both scores are likely attributable to the high pre-test probability in an operated cohort, whereas the relatively higher negative predictive value of RIPASA may help reduce missed diagnoses in similar high-prevalence settings.

### Knowledge gap and contribution

Prospective comparative data evaluating RIPASA and MAS using histopathological confirmation as the reference standard remain limited in Indian tertiary emergency settings. This study adds to existing evidence by providing clinically relevant estimates of diagnostic performance, including ROC-based discrimination with confidence intervals, and by highlighting the diagnostic trade-off between sensitivity and specificity at commonly used thresholds.

Strengths and limitations

Strengths of this study include prospective and consecutive enrolment, use of histopathology as the reference standard, prespecified and clinically relevant cut-offs, and comprehensive reporting of diagnostic performance.

However, several limitations should be considered. First, the study included only patients selected for appendicectomy, resulting in a high-prevalence operated cohort. This design may introduce spectrum and partial verification bias and may inflate positive predictive values, limiting generalizability to undifferentiated emergency department populations. Second, the modest sample size and the small number of negative appendicectomies (*n* = 6) resulted in wide confidence intervals for specificity and negative predictive value, reducing the precision of these estimates. Third, the single-centre design may limit external validity. Finally, interactions between clinical scores and imaging-based diagnostic pathways were not evaluated.

### Implications for practice and research

In comparable tertiary surgical settings—particularly where routine computed tomography (CT) is not readily available—RIPASA may help reduce missed diagnoses when used alongside clinical judgment and selective imaging. These findings should be interpreted within the context of operated cohorts and should not be generalized to undifferentiated emergency populations. Future multicentre studies are required to validate these findings across diverse populations and to evaluate integration with imaging-based diagnostic pathways.

## Conclusion

In this tertiary surgical cohort, RIPASA demonstrated higher sensitivity and better diagnostic discrimination than MAS, while MAS retained higher specificity. These findings suggest that RIPASA may help reduce missed diagnoses in similar high-prevalence emergency surgical settings. However, given the operated study population and modest sample size, further multicentre studies with broader undifferentiated patient populations are required before wider clinical generalisation.

## Data Availability

The de-identified dataset underlying the findings is available from the corresponding author on reasonable request.
